# Hospital Meetings

**Published:** 1904-03-05

**Authors:** 


### HOSPITAL MEETINGS.

### SEAMEN'S HOSPITAL SOCIETY.

The 83rd annual meeting of this society was held at the Institute of Chartered Accountants on the 26th ult., Mr. Percival Nairne presiding. The attention of the committee had, he said, been largely devoted during the year to modernising the hospital at Greenwich, and to the improvement and better equipment of the branch hospital at the docks. At the latter, the much-needed out-patient department was approaching completion, consisting of a waiting-room with accommodation for over fifty patients, a room for the out-patient physician or surgeon, an examining room, and a room for bandaging and minor injuries A new dispensary was attached, and for the first time adequate accommodation was provided for the dispenser. Over the out-patient department was an isolation block, with beds for two patients. A new kitchen had been provided, and a new mortuary. At the Dreadnought Hospital certain much-needed improvements had been effected. The kitchens had been newly equipped, the operating theatre improved, electric light had been installed, and a Roentgen-ray apparatus provided. The new light was greatly appreciated both by patients and staff, and was installed none too soon, for it was found that the gas pipes throughout the building were in a very defective condition. The total cost of this was £968 4s. 9d. All this required money, and no one acquainted with hospitals could fail to realise that the difficulty of raising funds was increased by the fact that special appeals were just now before the public from two or three of the large metropolitan hospitals. Without wishing for a moment to say anything which might limit the response to those appeals, he pointed out that the difficulty was a real one, and one that had to be faced. The Committee was very grateful for the help given to the hospital by the King's Fund, which had contributed in 1903 £1,000 as a special donation to the Tropical Diseases Department, with an additional £500 for that year, as well as the annual grant of £500. He added that it was very gratifying to notice the steady increase made by that fund, which was now being substantially assisted by the League of Mercy.

The London School of Tropical Medicine had progressed beyond the hopes or expectations of the founders, and the year had been one of much success. Acting on the recommendation of the School Committee, the Committee of Management agreed to the school being enlarged. The number of students had increased from an average of 25 to nearly 40, and the stay of each student had almost doubled. To provide adequately for this large number of post-graduates, the laboratory had been doubled in size, a museum and lecture theatre had been provided, additional sleeping accommodation afforded, and the mess-room much enlarged. These improvements had necessitated the expenditure of about £8,000, which sum had been advanced, by the Seamen's Hospital Society, at 4 per cent. The Hospital Committee had joined with the School Committee in requesting Sir Francis Lovell, whose mission to the tropics to collect funds for the School was so successful in 1901, to proceed again on a similar errand. He accordingly left England on February 9th, and the committee anticipated the greatest advantage to the school and hospital from his efforts. It was proposed to add his name to the Committee of Management. A very encouraging circumstance in the school's history was the visit, at Christmas, of the Paris School, when two professors and a dozen students went over the buildings. The recognition of the school by the London University, and the establishment of a Diploma of Tropical Medicine at Cambridge University, were matters for congratulation. The society was engaged in an eminently beneficent work, which was gaining the recognition not only of the Port of London, but of the world.

Sir Mark Young, in proposing the adoption of the report and accounts, remarked on the importance of the society from an Imperial point of view. Shipowners and others maintaining staffs in tropical climates could not fail to recognise the importance of keeping them at a high standard of efficiency, and to do this a knowledge of tropical disease was essential.

Captain Angove having seconded the motion, it was carried.

Mr. Swanzy, speaking with experience of West Africa, said the general health of the residents there had greatly improved, owing to the increased knowledge of the diseases to which that territory was liable, and he thought a larger degree of support should be contributed from that quarter. He regretted that his efforts to influence the members of the London Chamber of Commerce had not had the results for which he hoped.

The usual votes of thanks brought the meeting to a close.

### KING'S COLLEGE HOSPITAL.

The annual Court of Governors of this hospital was held on the 25th ult., Lord Methuen presiding.

The minutes of the special meeting of October 12th, at which the removal of the hospital to South London was sanctioned, were read and confirmed. The adoption of the report and accounts for 1903 was proposed by Mr. Charles Awdry, treasurer, who briefly referred to the removal. The choice of a site had occupied the attention of the committee, who, after the most careful investigations, had eventually selected an admirably placed estate of nearly 12 acres at Denmark Hill. This site the Hon. W. F. D. Smith, M.P., already a most generous benefactor of the hospital, had announced his intention of presenting to the Corporation, and it had been decided by the Removal Committee that the administrative block should be named in memory of his father, the Right Hon. W. H. Smith. The Committee of Management desired to place upon record their deep and lasting sense of gratitude for this most munificent gift. Considerable progress had been made in the collection of the sum of £300,000, estimated to be required for the removal of the hospital, upwards of £100,000, including the value of the site, having already been paid or promised. The project of removal had been warmly commended by the Committee of King Edward's Hospital Fund, who had made a grant of £5,000 towards the building fund. The Rev. A. C. Headlam having seconded the motion, it was adopted unanimously.

The name of the Bishop of Exeter, Dr. Robertson, formerly Principal of King's College, having been added to the list of vice-presidents, in recognition of his services as a member of the Management Committee, the officers for the ensuing year were re-elected, and the usual votes of thanks passed.

The Chairman said he had undertaken the office which he held in succession to Lord Dillon because of the high opinion he entertained of the work of the hospital. The task before the committee was an exceptionally difficult one, and the best service that could be rendered by the friends of the hospital was to let the truth as to the need for removal be as widely known as possible. It could not be too strongly impressed upon the public mind that the removal was an absolute necessity, both for the sake of the large population of South London, and for the greater efficiency of the hospital itself. Having briefly alluded to the forthcoming ball and tournament in aid of the funds, Lord Methuen concluded by assuring those present that the Committee intended to carry out to the full the intention of the Governors as expressed at the special meeting held in October.

In reply to the question put by one of the Governors present as to the future work of the medical school, the Rev A. C. Headlam (Chairman of the Removal Committee) said that as far as could be judged, the removal would be distinctly beneficial both to the hospital and the college. The London University was dividing the medical course into two parts—one confined to the hospital, the other apart from it. The preliminary scientific part of the curriculum would be fittingly carried on in the college, while the medical, surgical, and clinical instruction would be given in the hospital. This exactly corresponded with the views of the Governors in sanctioning the removal. He desired to draw attention to a meeting arranged by the Lord Mayor, to take place at the Mansion House on March 11th, at three o'clock, in support of the appeal for funds for the removal.

### CHARING CROSS HOSPITAL.

The chair was taken at the annual court of Governors of this hospital on February 24th by Mr. T. P. Borrett. In moving the adoption of the report he said that the first and largest half of the new buildings that had been begun in 1897 was now approaching completion and most people would allow that they were handsome and imposing. The nurses' home was replete with every comfort and convenience; each nurse had a separate bedroom. The additional buildings included four isolation wards and new quarters for the resident staff. He wished to pay a tribute to the admirable and uncomplaining manner in which both the nurses and the staff had performed their duties during the alterations. The new Levy ward had been opened last November when Miss Levy gave an additional sum of £3,000, and also most of the furniture for the new ward. Having referred in sympathetic terms to the death of Prebendary Kitto, he passed on to the question of finance. They had succeeded in selling the Chartlands Farm at a very good figure. The financial position of the hospital had always been a matter of anxiety to the Council and although subscriptions had increased they had a deficit on the year of £6,476, which made a total deficit at date of £44,593. The Council were negotiating a loan of £85,000

to be secured by a mortgage on the freehold of the hospital. He made a confident appeal to the generosity of the public to assist them to raise the money needed for maintenance and the completion of the new buildings, *i.e.* £36,000 in all.

The Earl of Kilmorey seconded the resolution and said that he would do all in his power to further the interests of the hospital in future.

The report for 1903 showed that 1,566 in-patients had been treated and 13,939 out-patients. In consequence of the closing of the hospital for three months owing to the old grand staircase being pulled down and a new one built, the number of patients treated was less than 1902. The total expenditure for the year was £13,660. The cost of each in-patient works out at £7 17s., and the cost per bed at £81 15s.

### NATIONAL ORTHOPÆDIC HOSPITAL.

The annual meeting of Governors was held at this hospital on February 26th. Letters of regret were read from the president (the Duke of Marlborough) and the treasurer (Lord Farquhar), who were unable to be present. The chair was taken by Mr. J. R. Cooper, chairman of the Executive Committee, who moved the adoption of the report for 1903, and said that it contained much that should be gratifying to the Governors. There was an increase of patients and also of subscriptions, and, although the expenditure was greater, yet the balance was on the right side. He was glad to be able to state that the forecast he had made in his speech at last year's meeting regarding amalgamation with the Royal Orthopædic Hospital had been fulfilled and the amalgamation would shortly be effected. This was the last meeting the National Orthopædic would hold with its Governors. The work done by the hospital during its career of 25 years had been one of uninterrupted success; from a very small institution it had grown till it was now to be ranked with the most powerful hospitals in London. Instead of 60 or 70 beds the new Orthopædic Hospital would have in future not less than 200 at the enlarged buildings in Great Portland Street and Bolsover Street, whilst there would be erected an outpost in the north of London which would accommodate 100 more. He ventured to think that a hospital with 300 beds would be regarded as one of the foremost institutions of its kind in London. Within a few weeks the National Orthopædic Hospital would be a thing of the past. They were glad to state that their staff, to whose services they were so much indebted, would constitute the staff of the new hospital, augmented by the staff of the Royal Orthopædic.

The resolution was seconded by Mr. S. N. Vigers and carried.

Later in the proceedings the chairman, in proposing a vote of thanks to the officers of the institution, paid a warm tribute to the splendid work done by the matron, and said they were all much gratified to think that their matron would be the matron of the new hospital, which fact they considered would contribute greatly to the success of the new institution.

The report showed that the receipts for 1903 were £3,302 against £2,728 in 1902, whilst expenditure figured as £3,511 against £3,045. The number of in-patients treated in 1903 was 245 and of out-patients 1,663, the figures in 1902 being 234 and 1,430.

The committee appealed earnestly for the sum of £60,000 required for rebuilding, as well as for an increase in annual subscriptions in order to render the amalgamated institutions a free hospital.

